# Murine analogues of etanercept and of F8-IL10 inhibit the progression of collagen-induced arthritis in the mouse

**DOI:** 10.1186/ar4319

**Published:** 2013-09-27

**Authors:** Fabia Doll, Kathrin Schwager, Teresa Hemmerle, Dario Neri

**Affiliations:** 1 Department of Chemistry and Applied Biosciences, Swiss Federal Institute of Technology (ETH Zürich), Wolfgang-Pauli-Strasse 10, CH-8093 Zurich, Switzerland; 2 Philochem AG, Libernstrasse 3, CH-8112 Otelfingen, Switzerland

## Abstract

**Introduction:**

Etanercept is a fusion protein consisting of the soluble portion of the p75-tumor necrosis factor receptor (TNFR) and the Fc fragment of human IgG1, which is often used for the treatment of patients with rheumatoid arthritis. F8-IL10 is a human immunocytokine based on the F8 antibody and interleukin-10, which is currently being investigated in rheumatoid arthritis with promising clinical results. We have aimed at expressing murine versions of these two fusion proteins, in order to assess their pharmaceutical performance in the collagen-induced model of rheumatoid arthritis in the mouse.

**Methods:**

Two fusion proteins (termed muTNFR-Fc and F8-muIL10) were cloned, expressed in chinese hamster ovary (CHO) cells, purified and characterized. Biological activity of muTNFR-Fc was assessed by its ability to inhibit TNF-induced killing of mouse fibroblasts, while F8-muIL10 was characterized in terms of muIL10 activity, of binding affinity to the cognate antigen of F8, the alternatively-spliced EDA domain of fibronectin, by quantitative biodistribution analysis and *in vivo* imaging. The therapeutic activity of both fusion proteins was investigated in a collagen-induced mouse model of arthritis. Mouse plasma was analyzed for anti-drug antibody formation and cytokine levels were determined by bead-based multiplex technology. The association of F8-IL10 proteins with blood cells was studied in a centrifugation assay with radiolabeled protein.

**Results:**

Both fusion proteins exhibited excellent purity and full biological activity *in vitro*. In addition, F8-muIL10 was able to localize on newly-formed blood vessels *in vivo*. When used in a murine model of arthritis, the two proteins inhibited arthritis progression. The activity of muTNFR-Fc was tested alone and in combination with F8-huIL10. The chimeric version of F8-IL10 was not better then the fully human fusion protein and showed similar generation of mouse anti-fusion protein antibodies. Incubation studies of F8-muIL10 and F8-huIL10 with blood revealed that only the fully human fusion protein is not associated with cellular components at concentrations as low as 0.2 μg/ml, thus facilitating its extravasation from blood vessels.

**Conclusions:**

The described products may represent useful research tools for the study of the anti-arthritic properties of TNF blockade and of IL10-based immunocytokines in syngeneic immunocompetent models of arthritis.

## Introduction

The therapeutic blockade of proinflammatory cytokines has revolutionized the treatment of rheumatoid arthritis (RA), leading to the approval and widespread use of biologics for this indication. The most prominent target is represented by tumor necrosis factor (TNF), a dominant cytokine capable of inducing the expression of other proinflammatory cytokines [1,2]. Infliximab (Remicade™), a blocking chimeric monoclonal antibody, and etanercept (Enbrel™), a fusion protein consisting of the soluble portion of the p75-TNF receptor (TNFR) and the Fc fragment of human IgG1 (hinge, CH2 and CH3 domain), were the first TNF-specific biological therapeutics to make a substantial impact for the treatment of RA. Later, adalimumab (Humira™, a fully human inhibitory antibody), certolizumab pegol (Cimzia™, a PEGylated humanized antibody Fab′ fragment) and golimumab (Simponi™, also a human blocking antibody) were approved. Other relevant proinflammatory cytokines include interleukin (IL)-1β (which is targeted by anakinra, a recombinant form of the IL-1 receptor antagonist) and IL-6 (whose action is blocked by tocilizumab, a monoclonal antibody specific to the IL-6 receptor) [3].

The use of anti-inflammatory cytokines represents an alternative approach for the treatment of RA, but has met with limited success until now. The most prominent example is represented by recombinant human IL-10, which has shown activity in the collagen-induced model of arthritis [4] and which has been investigated in controlled clinical studies [5]. A good safety profile, but only limited efficacy, was observed in patients receiving IL-10 (Tenovil™) for 28 days [6]. A combination of IL-10 and methotrexate was tested in a multicenter, placebo-controlled, dose-escalating study. The product was administered at a dose of 8 μg/kg four times daily or 8 μg/kg three times per week, with an American College of Rheumatology 20% (ACR20) improved response in 50% of the patients compared with 63% when treated with 20 μg/kg three times per week and only 10% in patients receiving placebo as control. However, American College of Rheumatology 50% (ACR50) improved benefits were less impressive, which probably led to the discontinuation of clinical development activities [5].

We recently reported the use of antibody-cytokine fusion proteins (immunocytokines) for the treatment of arthritis in the mouse. Particularly, we have shown that immunocytokines consisting of the L19 antibody or the F8 antibody (serving as delivery vehicle) and of human IL-10 were able to inhibit arthritis progression in the murine model of collagen-induced arthritis [7,8]. The L19 and F8 human antibodies recognize the alternatively-spliced extra-domain B(EDB) and extra-domain A(EDA) of fibronectin [9,10], respectively, which are not present in plasma fibronectin and which are almost undetectable in normal adult tissues. Both fusion proteins exhibited an ability to selectively localize at sites of arthritis *in vivo* and to stain pathological specimens in immunohistochemistry, while the reactivity to normal tissues was mainly confined to placenta and to the endometrium in the proliferative phase [8]. Based on these promising results, the F8-IL10 fusion protein was moved to a phase Ib clinical trial in patients with RA in combination with methotrexate. The study is still ongoing, but encouraging interim results have been reported [11].

As the combination of TNF blockade and recombinant IL-10 had previously exhibited encouraging results in the collagen-induced arthritis model [4], we became interested in studying whether a combination with F8-IL10 would also exhibit a potent inhibition of disease progression. For these preclinical studies it would be preferable to use reagents that display their full activity in the mouse. The clinically approved antibody-based products Remicade™, Humira™, Cimzia™ and Simponi™ exhibit little or no activity in the mouse as they display a much reduced affinity towards murine TNF compared with human TNF. By contrast, Enbrel™ is frequently used as a TNF blocker in mouse models of RA as it is active in blocking both human and murine TNF with similar activity [12].

A fusion protein consisting of the murine soluble portion of the p75-TNF receptor (amino acids 1 to 257) fused to murine IgG1 (termed by the authors murine p75-murine IgG1) has previously been reported in a short communication [13], but the full amino acid sequence of the product was not disclosed. The pharmacokinetic parameters of the murine p75-murine IgG1 fusion protein were studied in mice and were found to be different in healthy mice and mice with candidiasis, or compared with etanercept in humans [14]. No direct pharmacokinetic comparison between murine p75-murine IgG1 and etanercept was reported in the study.

To study the therapeutic potential of a combination of TNF blockade and F8-IL10, we here report on the cloning, expression and characterization of murine versions of etanercept (murine TNFR-Fc) and of F8-IL10 (F8-muIL10). The fusion proteins were studied both *in vitro* and *in vivo*. The murine version of etanercept exhibited an inhibition of arthritis progression when used alone or in combination with F8-IL10. Also F8-muIL10 inhibited arthritis progression, but not more efficiently than the fully human F8-IL10 counterpart. The new murine products may represent useful research tools for the study of TNF blockade and targeted cytokine delivery in mouse models of RA and other inflammatory conditions. Surprisingly, we observed a different ability of F8-muIL10 and of F8-huIL10 to interact with blood cells. Only the fully human fusion protein was not trapped by cellular components at concentrations as low as 0.2 μg/ml, thus facilitating its extravasation from blood vessels and its disease-targeting activity.

## Methods

### Cell lines and animals

CHO-S cells (Invitrogen, Zug, Switzerland) were cultured adherent in RPMI 1640 (Gibco, Zug, Switzerland) supplemented with 10% fetal bovine serum (Gibco), 2 mM ultraglutamine (Lonza, Basel, Switzerland) and antibiotics/antimycotics (Gibco) or in suspension in PowerCHO-2CD (Lonza) with 8 mM ultraglutamine, HT supplement (Gibco) and antibiotics/antimycotics in shaker incubators. Lung murine fibroblasts (CCL-1.3; ATCC, Molsheim Cedex, France) were cultured adherent in Dulbecco’s modified Eagle’s medium (Gibco) with 10% fetal bovine serum and antibiotics/antimycotics. MC/9 cells (murine mast cells, CRL-8306; ATCC) were cultured according to the supplier’s protocol in Dulbecco’s modified Eagle’s medium supplemented with 10% fetal bovine serum, 2 mM ultraglutamine, 10% rat-T-STIM (BD Becton Dickinson, Allschwil, Switzerland) and 0.05 mM β-mercaptoethanol (Gibco). Murine F9 teratocarcinoma cells (CRL-1720; ATCC) were cultured on 0.1% gelatin-coated tissue flasks in Dulbecco’s modified Eagle’s medium supplemented with 10% fetal bovine serum and antibiotics/antimycotics. Male DBA/1J mice were obtained from Janvier (Le Genest-St-Isle, France). Female 129/SvEv mice were obtained from Charles River (Sulzfeld, Germany).

### Cloning of murine fusion proteins

For cloning of muTNFR-Fc the murine TNFR gene (extracellular domain of TNFRII, amino acids 23 to 258) was amplified from previously cloned F8(scFv)-TNFRII [8] by polymerase chain reaction (PCR) using the primer pair 5′-CCTGTTCCTCGTCGCTGTGGCTACAGGTGTGCACTCGGTGCCCGCCCAGGTTGTCTT-3′ and 5′-CAATCCCTGGGCACGCCACCCTTGGTACTTTGTTC-3′ appending part of a signal sequence at the N-terminus and an overlapping fragment to muFc at the C-terminus. The gene for murine Fc (hinge, CH2, CH3; amino acids 98 to 324) was amplified from a commercial cDNA (Source BioScience, Berlin, Germany) using the primer pair 5′-CCAAGGGTGGCGTGCCCAGGGATTGTGGTTGTAAGC-3′ and 5′-TTTTCCTTTTGCGGCCGCTCATTAAGCTATTTACCAGGAGAGTGGGAGAGG-3′ appending an overlapping fragment to muTNFR at the N-terminus and a stop codon and *Not*I restriction site to the C-terminus. muTNFR and muFc sequences were PCR-assembled using the primer pair 5′-CCCAAGCTTGTCGACCATGGGCTGGAGCCTGATCCTCCTGTTCCTCGTCGCTGTGGC-3′ and 5′-TTTTCCTTTTGCGGCCGCTCATTAAGCTATTTACCAGGAGAGTGGGAGAGG-3′ appending the second part of the signal sequence and a *Hind*III restriction site to the N-terminus. The assembled fragment was double digested with *Hind*III/*Not*I (New England BioLabs, Allschwil, Switzerland) and ligated into the mammalian cell-expression vector pcDNA3.1(+) (Invitrogen) (for full sequence see Additional file 1).

F8-muIL10 was cloned using the sequence for F8 in diabody format [10] with the primers 5′-CCTGTTCCTCGTCGCTGTGGCTACAGGTGTGCACTCGGAGGTGCAGCTGTTGGAGTCTGGG-3′ and 5′-GATGAGCCGGAAGAGCTACTACCCGATGAGGAAGATTTGATTTCCACCTTGGTCCCTTGGCCGAA-3′ introducing part of a signal sequence at the N-terminus and part of a C-terminal (SSSSG)_3_ linker. The sequence for murine IL10 (amino acids 19 to 178) was amplified from a commercial cDNA (Sino Biological Inc., Beijing, China) using the primer pair 5′-GGGTAGTAGCTCTTCCGGCTCATCGTCCAGCGGCAGCAGGGGCCAGTACAGCCGGG-3′ and 5′-TTTCCTTTTGCGGCCGCCTAGCTTTTCATTTTGATCATCA TG-3′ appending a complementary part of the (SSSSG)_3_ linker at the N-terminus and a stop codon and *Not*I restriction site to the C-terminus. F8 diabody and murine IL-10 sequences were PCR-assembled using the primers 5′-CCCGCTAGCGTCGACCATGGGCTGGAGCCTGATCCTCCTGTTCCTCGTCGCTGTGGC-3′ and 5′-TTTCCTTTTGCGGCCGCCTAGCTTTTCATTTTGATCATCATG-3′ adding the rest of the signal sequence and a *Nhe*I restriction site to the N-terminus. The assembled fragment was double digested with *Nhe*I/*Not*I (New England BioLabs) and ligated into the mammalian cell-expression vector pcDNA3.1(+) (for full sequence see Additional file 2).

### Expression, purification and characterization of murine fusion proteins

The fusion proteins were expressed in a stable monoclonal cell line as reported before [15]. Briefly, PEI-mediated transient gene expression [15] was used to generate a polyclonal batch of protein. An aliquot of the transient gene expression culture was used to produce a stable cell line using geneticin (G418, 0.5 g/l; Santa Cruz, Heidelberg, Germany) for selection. Monoclonal cells were screened for high expression of protein by ELISA, using L19-TNFα (produced in our laboratory) as coating antigen and a goat anti-mouse IgG (Fc-specific)-peroxidase antibody (Sigma-Aldrich, Buchs, Switzerland) for detection in the case of muTNFR-Fc. For F8-muIL10, recombinant EDA was used as antigen and protein A–horseradish peroxidase (GE Healthcare, Glattbrugg, Switzerland) for detection. The best producing clone for each construct was grown in PowerCHO-2CD medium in suspension for large-scale production of protein. The proteins were purified from cell culture supernatant by protein A affinity chromatography and analyzed by SDS-PAGE, size exclusion chromatography (Superdex200 10/300GL; GE Healthcare) and for F8-muIL10 additionally by surface plasmon analysis (BIAcore) on an EDA-coated CM5 sensor chip (GE Healthcare).

### Bioactivity assays

The biological activity of muTNFR-Fc was determined by its ability to inhibit TNFα-induced killing of mouse fibroblasts [16]. Lung murine fibroblast cells were seeded in a 96-well plate (30,000 cells/well) in 100 μl culture medium and incubated for 24 hours at 37°C, 5% CO_2_. Medium containing actinomycin D (final concentration 2 μg/ml; Sigma-Aldrich), TNFα (final concentration of trimer 5 pM; eBioscience, Vienna, Austria) and different concentrations of muTNFR-Fc (serially diluted from 50 nM to 0.1 pM) was added to the cells. After incubation at 37°C for 24 hours cell viability was determined by addition of 20 μl Cell Titer Aqueous One Solution (Promega, Dübendorf, Switzerland), and after 2 hours absorption was measured at 490 nm.

For determination of the biological activity of the IL-10 moiety in the F8-muIL10 fusion protein, an IL-4-dependent proliferation assay of MC/9 cells was used [8]. Cells were seeded in a 96-well plate (40,000 cells/well) with 200 μl culture medium (without rat-T-STIM) containing 5 pg (0.05 units)/ml murine IL-4 (eBioscience) and varying concentrations of F8-muIL10 or recombinant murine IL-10 (eBioscience) starting at a concentration of 100 ng/ml IL-10 equivalents. After incubation at 37°C for 48 hours 20 μl/well Cell Titer Aqueous One Solution was added, and after 2 hours absorption was measured at 490 nm.

### Biodistribution

The *in vivo* targeting of F8-muIL10 was tested by quantitative biodistribution analysis using radiolabeled protein as described before [17]. For this analysis 129/SvEv mice were implanted subcutaneously (s.c.) with F9 tumor cells (25 × 10^6^ cells) in the flank. Purified F8-muIL10 (15 μg/mouse) was radioiodinated with ^125^I and injected intravenously (i.v.) into the lateral tail vein of mice (*n* = 3) grafted with F9 tumors. Mice were sacrificed 24 hours after injection. Organs were excised, weighed and radioactivity was counted using a Cobra γ counter (Packard Instrument Company, Meriden, CT, USA). Radioactivity content of representative organs was expressed as percentage of injected dose per gram of tissue.

### *In vivo* imaging

To test the targeting properties of the murine and human F8-IL10 fusion proteins, a near-infrared fluorescence imaging study was performed. For this purpose, the proteins (11 nmol F8-muIL10 and F8-huIL10) were incubated for 1 hour with a 20× molar excess of IRDye 750 N-hydroxysuccinimidyl ester (220 nmol; LI-COR, Bad Homburg, Germany) in 10% dimethylsulfoxide/phosphate-buffered saline (PBS), pH 7.4, at room temperature. Protein was purified from free dye using a PD10 desalting column (GE Healthcare), eluted in 5% dimethylsulfoxide/PBS and concentrated to 1.3 mg/ml using Amicon Ultra (10K) centrifugal filter units (Millipore, Zug, Switzerland). Then 200 μg (or 100 μg) of each protein were injected i.v. into the lateral tail vein of mice (*n* = 1) that had developed arthritis after the second collagen immunization (see section Mouse model of collagen-induced arthritis for more details). Mice were imaged at 1, 4, 24 and 48 hours after the injection under isoflurane anesthesia on their ventral side using an IVIS Spectrum machine (Xenogen, Caliper Life Sciences, Oftringen, Switzerland) with the following imaging parameters: λ_ex_ = 745 nm, λ_em_ = 800 nm, exposure time = 1 second, F/stop = 4, small binning. After 48 hours, mice were sacrificed and paws (arthritic and not affected ones) were photographed and then submitted to fluorescence imaging, using the same parameters.

### Mouse model of collagen-induced arthritis

Male DBA/1J mice (8 weeks old) were immunized by subcutaneous injection at the base of the tail with 0.05 ml emulsion of bovine type II collagen emulsified in Complete Freund’s Adjuvant (Hooke Laboratories, Lawrence, MA, USA). Three weeks later, a booster injection of 0.05 ml bovine collagen/Complete Freund’s Adjuvant in the case of the full collagen induction protocol and 0.04 ml for the reduced collagen induction protocol was given to the mice. After the booster injection, mice were inspected daily and disease was monitored using two different scoring systems. To each limb a clinical score was assigned (0 = normal, 1 = swelling of one or more toes of the same limb and 2 = swelling of the whole paw). A maximum score of eight can be reached in this first scoring system [8,18]. A more diverted clinical score, the modified score, was also used (0 = normal; 1 = one toe inflamed and swollen; 2 = more than one toe, but not entire paw, inflamed and swollen or mild swelling of entire paw; 3 = entire paw inflamed and swollen; 4 = very inflamed and swollen paw; adapted from Hooke Laboratories). A maximum score of 16 can be reached. In addition swelling of affected paws was measured daily with a caliper under isoflurane anesthesia. Paw thickness is expressed as the mean of all four paws of each animal. Animals were included into a therapy group when showing signs of joint inflammation with a score of 1 to 3. When the joint inflammation was too strong at day 1 (more than one paw, score >3) mice were not included into the experiment, because according to our project license (208/2010) we are not allowed to keep a mouse alive with a conventional arthritic score ≥4 for more than 4 days. All animal experiments were performed in agreement with Swiss ethical regulations. Ethical approval for all experiments was given by the state veterinary office (reference number 208/2010; Veterinäramt des Kantons Zürich, Zürich, Switzerland).

### Combination therapy of muTNFR-Fc and F8-huIL10

Mice were immunized according to the full collagen induction protocol. Mice with a new clinical score of 1 to 3 were randomly assigned to a treatment or control group and therapy was started (day 1). Mice received intravenous injections of muTNFR-Fc (10 μg) into the lateral tail vein or subcutaneous injections of F8-huIL10 (200 μg) or saline or a combination of muTNFR-Fc (10 μg, i.v.) and F8-huIL10 (200 μg, s.c.), three times on days 1, 4 and 7. Seven mice were analyzed per group in a daily, nonblinded fashion and the arthritic clinical score, the thickness of inflamed paws and weight was monitored. Mice were sacrificed at day 5 (PBS), day 8 (F8-huIL10, muTNFR-Fc) or day 13 (combination) due to arthritic score (≥4 for more than 4 days with conventional arthritic score) and weight loss (>15%), in accordance with local regulations.

### Comparison of F8-huIL10 and F8-muIL10

Mice were immunized according to the reduced collagen induction protocol. Therapy was performed as described before. Mice received either intravenous injections of muTNFR-Fc (30 μg) or subcutaneous injections of F8-huIL10 (200 μg), F8-muIL10 (200 μg) or saline or a combination of muTNFR-Fc (30 μg, i.v.) and F8-huIL10 (200 μg, s.c.). Ten mice were analyzed per group in a daily, nonblinded fashion and the arthritic clinical score, the thickness of inflamed paws and weight was monitored. Mice were sacrificed at day 8 (PBS), day 9 (F8-huIL10, muTNFR-Fc, F8-muIL10) or day 13 (combination) due to arthritic score and weight loss, in accordance with local regulations.

### Detection of anti-F8-huIL10/anti-F8-muIL10 antibodies in plasma of treated mice

Blood was obtained at the start of the therapy (day 1, *n* = 4) from the vena saphena or at the end of therapy from sacrificed mice through cardiac puncture, processed to plasma and stored at -20°C. The immunogenicity of F8-huIL10 and F8-muIL10 was assessed by surface plasmon resonance (BIAcore 3000) screening of mouse plasma samples. F8-huIL10 or F8-muIL10 at a concentration of 50 μg/ml were immobilized on a CM5 sensor chip (GE Healthcare) using an amine coupling kit (GE Healthcare). Surface density of 2,600 RU and 2,900 RU was achieved for F8-huIL10 and F8-muIL10, respectively. On a control flow cell, activation by 1-ethyl-3-(3-dimethylaminopropyl)carbodiimide hydrochloride (EDC) and N-hydroxysuccinimide (NHS) was performed and immediately blocked by injecting ethanolamine. For binding analysis, positive control samples of anti-human IL-10 and anti-murine IL-10 antibodies (1 and 4 μg/ml; eBioscience) and serum samples diluted 500-fold in HBS-EP buffer (GE Healthcare) were passed over the different flow cells with a flow rate of 30 μl/minute for 3 minutes. The response was recorded 30 seconds after the end of the injection. The positive control was run again at the end of the analysis to confirm binding capacity of the immobilized protein. To regenerate the surface, 10 mM glycine, pH 2.0, was run over the flow cells for 40 seconds at 30 μl/minute.

### Analysis of cytokine levels in plasma of mice

Blood was obtained at the end of therapy from each mouse (see above), processed to plasma and stored at -20°C. To quantify cytokine levels in plasma of treated and control mice, a multiplex bead-based flow cytometry analysis was performed using the Mouse Th1/Th2/Th17/Th22 13plex FlowCytomix Multiplex (eBioscience) following the supplier’s protocol. Fluorescence-activated cell sorting analysis was performed on a BD FACS Canto (BD Bioscience, Allschwil, Switzerland) and data evaluated with FlowCytomix Pro 3.0 software (eBioscience). The experiment was repeated on a different day in order to have an independent replicate of the assay (Additional files 3 and 4). Using standard curves generated by the FlowCytomix Pro 3.0 software with positive control samples, a level of quantification was assigned to every cytokine (Additional files 5 and 6).

### Incubation experiment of radiolabeled immunocytokines with whole blood

The ability of F8-huIL10 and F8-muIL10 to interact with blood cells was determined by a centrifugation-based assay with radiolabeled preparations. Purified F8-huIL10 and F8-muIL10 were radioiodinated with ^125^I as described before [17] and different concentrations of labeled protein were incubated with fresh human and mouse blood. Human blood was collected in S-Monovette tubes (Kalium-EDTA (ethylenediamine tetraacetic acid), Sarstedt, Sevelen, Switzerland) and mouse blood taken from DBA/1J mice via cardiac puncture after sacrifice using Microtainer LH tubes (lithium heparin, BD Bioscience) to prevent coagulation. After 10 minutes of incubation, tubes were centrifuged for 3 minutes at 2,000 × *g*. Plasma was separated from the cell pellet and radioactivity of both was counted using a Cobra γ counter.

### Statistical analysis

Data are expressed as the mean ± standard deviation or standard error of the mean. Differences in arthritic outcome between therapeutic groups were compared using GraphPad Prism (GraphPad Software Inc., La Jolla, CA, USA) grouped two-way ANOVA multiple-comparison (Bonferroni-corrected) analysis, with *P* <0.05 considered significant. Differences in cytokine levels were compared using a Mann–Whitney test, with *P* <0.05 considered significant.

## Results

### Cloning, expression and characterization of fusion proteins

**Figure 1:**
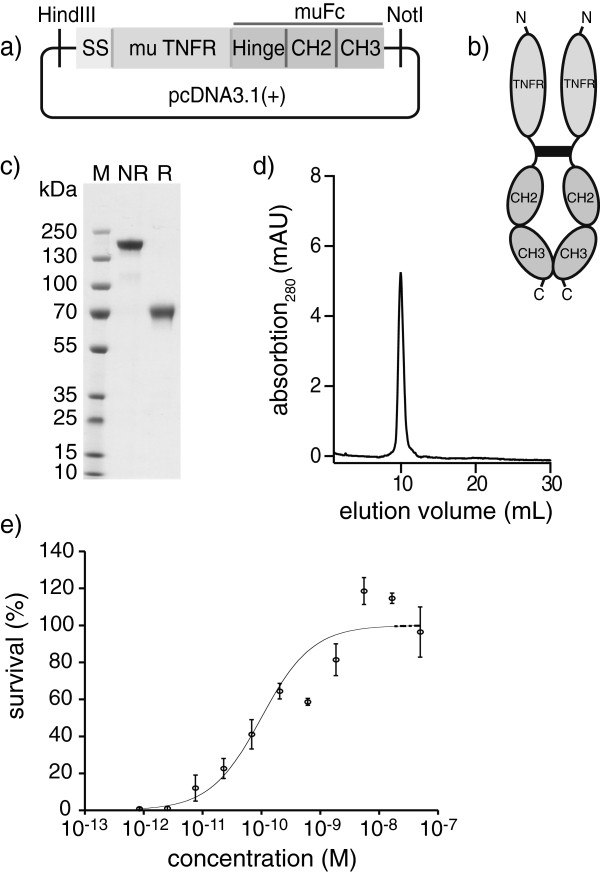
**Cloning, expression and *in vitro *characterization of muTNFR-Fc. (a)** Schematic representation of the cloning strategy of muTNFR-Fc. muTNFR was directly fused to the Fc fragment (hinge, CH2 and CH3 regions) of murine IgG1, containing a signal sequence (SS) for secretion of the protein at the N-terminus. **(b)** Schematic representation of the formation of a muTNFR-Fc dimer trough disulfide bridges in the hinge region. **(c)** SDS-PAGE analysis of purified muTNFR-Fc. M, molecular weight marker; NR, nonreducing conditions; R, reducing conditions. **(d)** Size exclusion chromatography (SEC200) of covalent homodimeric muTNFR-Fc. **(e)** Bioactivity assay of muTNFR-Fc. muTNFR-Fc inhibited tumor necrosis factor (TNF)-induced killing of mouse fibroblasts with a half-maximal inhibitory concentration of 0.1 nM (mean ± standard deviation, *n* = 3).

The murine fusion protein muTNFR-Fc was cloned and expressed by stable transfection in CHO cells, appending the extracellular part (amino acids 23 to 258) of the murine p75-TNF receptor at the N-terminus of a murine IgG1 Fc portion, containing the hinge region (Figure 1a,b). A complete sequence of muTNFR-Fc is reported in Additional file 1. The fusion protein was purified from the culture supernatant by protein A chromatography, yielding a preparation that was pure in SDS-PAGE analysis and size exclusion chromatography (Figure 1c,d). The biological activity of muTNFR-Fc was tested by inhibition of TNF-mediated killing of lung murine fibroblasts [16], exhibiting a half-maximal inhibitory concentration value of 0.1 nM (Figure 1e).

**Figure 2:**
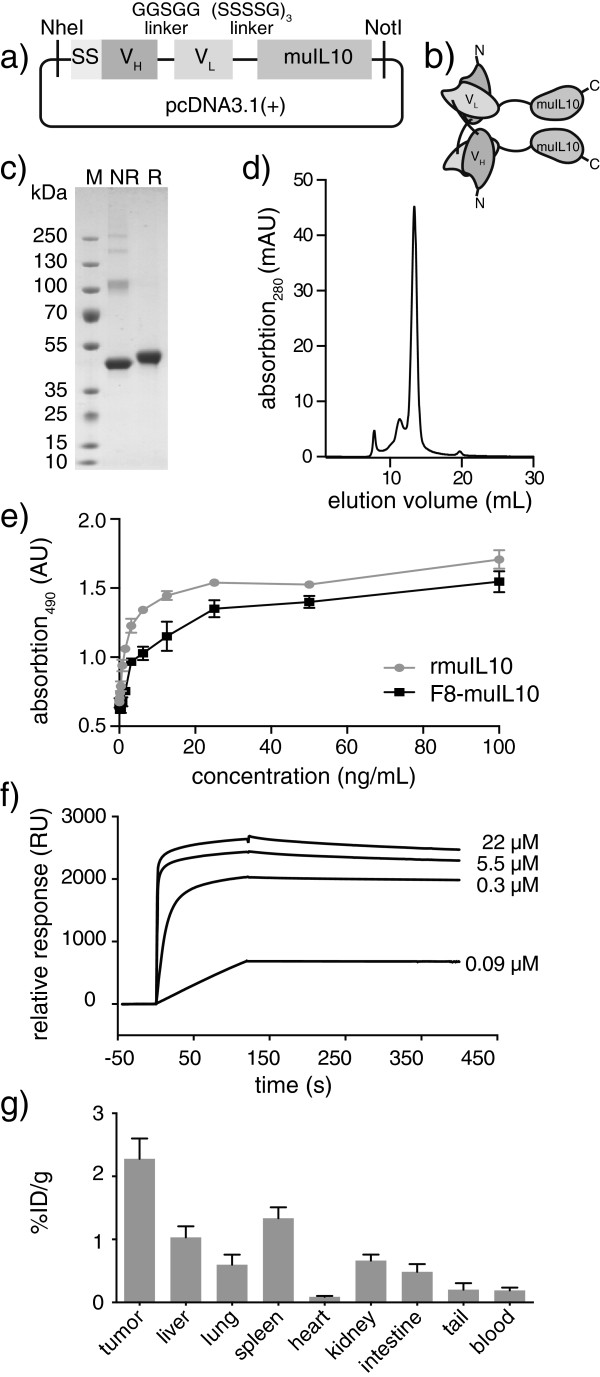
**Cloning, expression and *in vitro *characterization of F8-muIL10. (a)** Schematic representation of the cloning strategy of F8-muIL10. The murine IL-10 moiety was fused by a 15-amino-acid linker (SSSSG)_3_ to the C-terminus of the F8 scFv antibody fragment in diabody format (five-amino-acid linker between variable heavy chain (V_H_) and variable light chain (V_L_)). SS, signal sequence. **(b)** Schematic representation of protein domain assembly of the noncovalent F8-muIL10 dimer. **(c)** SDS-PAGE analysis of purified F8-muIL10. M, molecular weight marker; NR, nonreducing conditions; R, reducing conditions. **(d)** Size exclusion chromatography (SEC200) of noncovalent homodimeric F8-muIL10. **(e)** MC/9 cell proliferation assay. F8-muIL10 and recombinant murine IL10 induced proliferation of MC/9 cells (mean ± standard deviation (SD), *n* = 3). **(f)** BIAcore analysis of F8-muIL10 on extra-domain A of fibronectin (EDA)-coated chip. **(g)** Quantitative biodistribution study of radioiodinated F8-muIL10. Mice bearing subcutaneous F9 tumors were injected intravenously with 15 μg radiolabeled protein (*n* = 3). Mice were sacrificed after 24 hours and organs were excised and radioactivity counted, expressing results as percent of injected dose per gram of tissue (%ID/g ± SD).

The production and characterization of the fully human fusion protein F8-huIL10 have previously been described by our group [8]. In addition, the fusion protein F8-muIL10 was cloned and expressed by stable transfection in CHO cells. Murine IL-10 was appended at the C-terminus of the F8 antibody in noncovalent homodimeric scFv (diabody) format [10], using a five-amino-acid linker between variable heavy chain (V_H_) and variable light chain(V_L_), and a 15-amino-acid linker between the antibody and muIL10 (Figure 2a,b). A complete sequence of F8-muIL10 is reported in Additional file 2. Also in this case, the fusion protein was purified by protein A chromatography from the cell culture supernatant, yielding a well-behaved protein preparation (Figure 2c,d). The biological activity of F8-muIL10 was tested by a proliferation assay on MC/9 cells, as previously described [8] (Figure 2e). Furthermore, the formation of a high-affinity and kinetically stable complex between F8-muIL10 and its cognate antigen (the alternatively spliced EDA domain of fibronectin) was confirmed by BIAcore analysis (Figure 2f). *In vivo* targeting was assessed by quantitative biodistribution using radioiodinated F8-muIL10 injected i.v. into mice bearing subcutaneous F9 tumors. Selective tumor targeting was seen, with a tumor to blood ratio of 12, but spleen and liver uptake were also high (Figure 2g).

**Figure 3:**
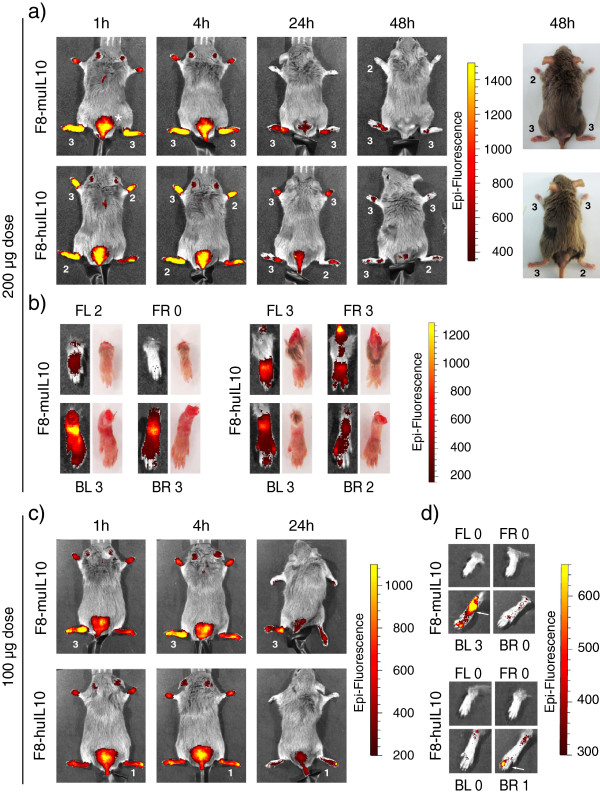
***In vivo *near-infrared fluorescence imaging of F8-IL10 fusion proteins in mice with collagen-induced arthritis.** Arthritic mice (*n* = 1) were injected intravenously with 200 or 100 μg F8-muIL10 or F8-huIL10 labeled with IRDye 750. **(a)** Mice injected with 200 μg of fusion protein were imaged 1, 4, 24 and 48 hours after injection. **(b)** After 48 hours mice were sacrificed and individual paws were imaged. In addition, photographs of paws are shown to illustrate the paw swelling. **(c)** Mice injected with 100 μg fusion protein were imaged 1, 4, and 24 hours after injection. **(d)** After 24 hours mice were sacrificed and individual paws were imaged. Indicated numbers represent the score of the according paw: 1, one toe inflamed and swollen; 2, more than one toe, but not entire paw, inflamed and swollen or mild swelling of entire paw; 3, entire paw inflamed and swollen. FL, front left; FR, front right; BL, back left; BR, back right; plus the according score. *Site of immunization.

In addition the *in vivo* targeting properties of F8-muIL10 and F8-huIL10 were investigated in mice with collagen-induced arthritis, using near-infrared fluorescence imaging. The fusion proteins, studied at 100 μg and 200 μg doses after labeling with IRDye 750, were injected i.v. and mice were imaged 1, 4, 24, and 48 hours after injection, always using the same acquisition parameters. The experiments showed a preferential accumulation of the F8-IL10 fusion proteins in inflamed paws and toes, which slowly declined over time (Figure 3).

### Therapy experiments in the collagen-induced model of arthritis

**Figure 4:**
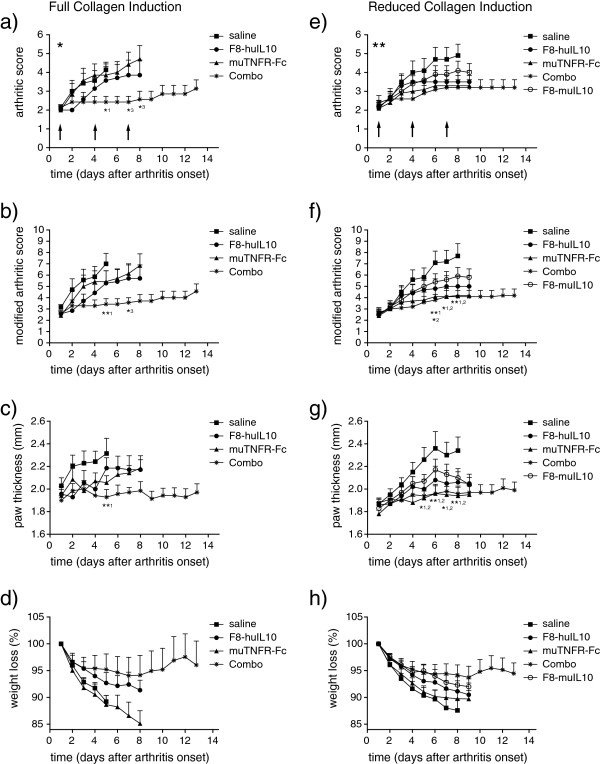
**Therapy studies in mice with collagen-induced arthritis. (a) to (d)** Mice were immunized according to the full collagen induction protocol, included in the therapy when showing symptoms and injected on days 1, 4 and 7 (arrows). *Fifteen of 50 mice (30%) developed an explosive disease and could not be included in the therapy. Mice received saline subcutaneously (s.c.; squares), 200 μg F8-huIL10 s.c. (circles), 10 μg muTNFR-Fc intravenously (i.v.; triangles) or a combination of F8-huIL10 and muTNFR-Fc (crosses). The clinical score was evaluated daily and expressed as **(a)** arthritic score or **(b)** modified arthritic score with mean and standard error of the mean (SEM) of seven mice per group. **(c)** Paw swelling was measured daily and paw thickness expressed as the mean of all four paws (mean and SEM). **(d)** Weight was monitored daily and expressed as percent of weight loss (mean and SEM). **(e) to (h)** Mice were immunized according to the reduced collagen induction protocol. **Six of 60 mice (10%) developed disease too rapidly and could not be included in the therapy. Mice received saline s.c. (squares), 200 μg F8-huIL10 s.c. (circles), 30 μg muTNFR-Fc i.v. (triangles), a combination of F8-huIL10 and muTNFR-Fc (crosses) or 200 μg F8-muIL10 s.c. (open circles). **(e)** Arthritic score or **(f)** modified arthritic score with mean and SEM of 10 mice per group. **(g)** Paw swelling was measured daily and paw thickness expressed as the mean of all four paws (mean and SEM). **(h)** Weight was monitored daily and expressed as percent of weight loss (mean and SEM). *^1^*P* <0.05, **^1^*P* <0.01 combo versus saline; *^2^*P* <0.05, **^2^*P* <0.01 muTNFR-Fc versus saline; *^3^*P* <0.05 combo versus muTNFR-Fc.

The therapeutic activity of muTNFR-Fc and of F8-IL10 proteins (F8-huIL10 and F8-muIL10) was tested in the collagen-induced model of rheumatoid arthritis in male DBA/1J mice. In a first experiment, we used a full collagen induction (0.05 ml emulsion of bovine type II collagen emulsified in Completes Freund’s Adjuvant and a booster injection of 0.05 ml of the same emulsion), which led to a rapid development of the disease. Arthritis severity was monitored using a conventional arthritic score (1 point for each limb with at least one affected toe, 2 points if the whole paw is swollen) and a modified score (1 point if one toe per limb is affected, 2 points if more than one toe per limb is affected or a moderate paw swelling is observed, 3 points if the entire paw is swollen, 4 points if the paw is severely swollen). Treatment was started when mice displayed an arthritic score of 1, 2 or 3 (Figure 4). However, out of 50 mice scheduled for treatment, 15 (30%) developed an explosive disease (moved within 1 day from score 0 to score >3) and could not be included in the therapy experiment. The remaining mice were treated with three injections of saline (s.c.), muTNFR-Fc (10 μg, i.v.), F8-huIL10 (200 μg, s.c.) or the combination of muTNFR-Fc and F8-huIL10. The strongest inhibition of arthritis progression (both in terms of arthritic score and of paw swelling) was observed for the combination treatment (*P* <0.01 vs. saline), whereas the single agents did not display a significant activity in this aggressive model of arthritis with full collagen induction (Figure 4a,b,c).

We repeated the experiment using a reduced collagen induction schedule, featuring a reduced booster injection (reduction to 80%, 0.04 ml emulsion instead of 0.05 ml). In this case, only 6/60 mice (10%) could not be included into the therapy experiment, because they progressed too rapidly from score 0 to score >3. F8-muIL10 (200 μg s.c.), F8-huIL10 (200 μg, s.c.) and muTNFR-Fc (30 μg, i.v.; alone or in combination with F8-huIL10) displayed an inhibition of disease progression compared with saline treatment (Figure 4e,f,g), being statistically significant for muTNFR-Fc alone or in combination with F8-huIL10 compared with saline from day 6 onwards (*P* <0.05). Over time, mice lost weight because of arthritis progression, an effect that was reduced by the combination therapy (Figure 4d,h). The administration of IL-10 fused to an antibody of irrelevant specificity in the mouse (HyHel10-IL10) has previously been described, showing inhibition activities that were lower than the corresponding fusion proteins based on the F8 or L19 antibodies [7,8].

### Characterization of mouse plasma

**Figure 5:**
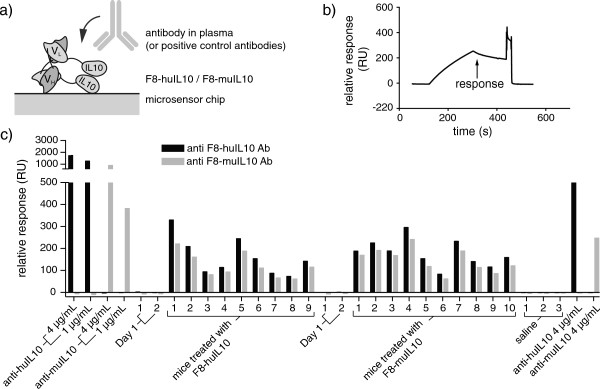
**Analysis of mouse anti-fusion protein antibody response. (a)** Schematic representation of the BIAcore experiment. F8-huIL10 or F8-muIL10 was immobilized on a microsensor chip. Plasma of mice treated with either the fully human F8-huIL10 or the chimeric F8-muIL10 fusion protein or positive control antibodies (anti-huIL10 or anti-muIL10) were passed over the different flow cells. **(b)** The flow rate over the sensor surface was 30 μl/minute for 3 minutes and the response was recorded 30 seconds after the end of the injection. **(c)** Relative response (RU) of plasma samples and positive controls. Samples were passed over the two different flow cells coated with F8-huIL10 (black bars) or F8-muIL10 (grey bars) and binding of mouse anti-fusion protein antibody (MAFA) expressed as relative response. Ab, antibody.

Plasma was collected from mice before the first therapeutic injection and when they were sacrificed. We used BIAcore technology in order to assess whether mice developed a mouse anti-fusion protein antibody (MAFA) response as a result of treatment with the fully human F8-huIL10 or the chimeric F8-muIL10 fusion protein (Figure 5a,b). Plasma samples were studied at a dilution of 1:500, whereas monoclonal antibodies specific to human and murine IL-10 were used as positive controls in this assay at concentrations of 1 and 4 μg/ml. Plasma samples before immunocytokine treatment (day 1) did not display a detectable BIAcore response, whereas all samples at the end of treatment revealed the presence of a MAFA reaction. In this assay, there was no significant difference between the use of the fully human F8-huIL10 or of the chimeric F8-muIL10 fusion protein, suggesting that the main immunogenic contribution in the mouse was due to the human F8 antibody moiety (Figure 5c). An assessment of immunogenicity based on binding velocity on BIAcore chips, rather than on BIAcore response units at saturation, did not reveal a substantial difference for the quantification of mouse anti-fusion protein antibodies (Additional file 7).

**Figure 6:**
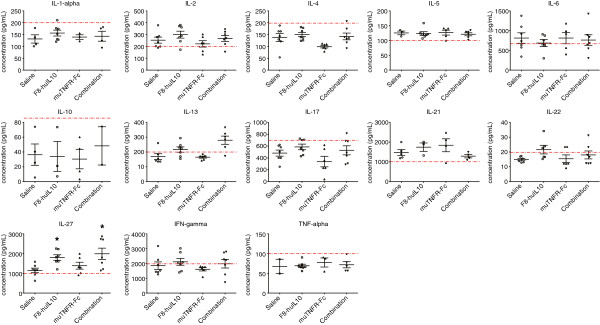
**Analysis of cytokine levels in mice with full collagen-induced arthritis.** At the end of the therapy 13 different cytokine concentrations were measured in plasma using multiplex bead-based flow cytometry. Data points of cytokine concentrations above detection level are represented in a scatter plot with the mean ± standard error of the mean (*n* = 7). Standard curves defined with positive control samples (Additional file 5) were used to generate a level of quantification (LOQ; dotted red line). Most of the measured concentrations were below the LOQ, but a significant increase in the level of IL-27 was observed for F8-huIL10 and the combination versus saline (*P = 0.0017 and *P* = 0.038, respectively). IFN, interferon; IL, interleukin; TNF, tumor necrosis factor.

**Figure 7:**
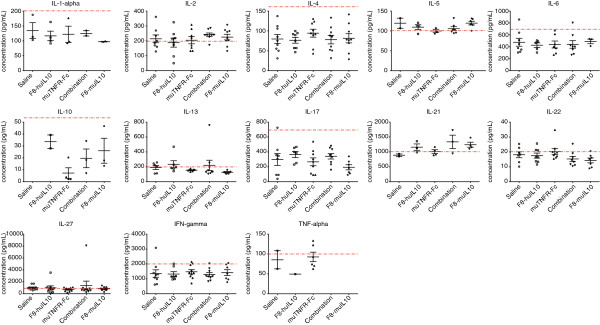
**Analysis of cytokine levels in mice with reduced collagen-induced arthritis.** At the end of the therapy 13 different cytokine concentrations were measured in plasma using multiplex bead-based flow cytometry. Data points of cytokine concentrations above detection level are represented in a scatter plot with the mean ± standard error of the mean (*n* = 10). Standard curves defined with positive control samples (Additional file 5) were used to generate a level of quantification (LOQ; dotted red line). IFN, interferon; IL, interleukin; TNF, tumor necrosis factor.

Mouse plasma was also used to measure cytokine levels using a multiplex bead-based assay. The results of these measurements are reported in Figures 6 and 7, corresponding to the therapy experiments with full collagen induction and reduced collagen induction. For each sample and cytokine, measurements were repeated on a different day in order to have an independent replicate of the assay (Additional files 3 and 4). Using standard curves defined with positive control samples (Additional file 5) a level of quantification was assigned to every cytokine. Most of the measured concentrations were below the level of quantification, but a significant increase in the level of IL-27 was observed for F8-huIL10 and the combination versus saline (*P* = 0.0017 and *P* = 0.038, respectively) in the full collagen induction setting.

### Incubation of immunocytokines with whole blood

**Figure 8:**
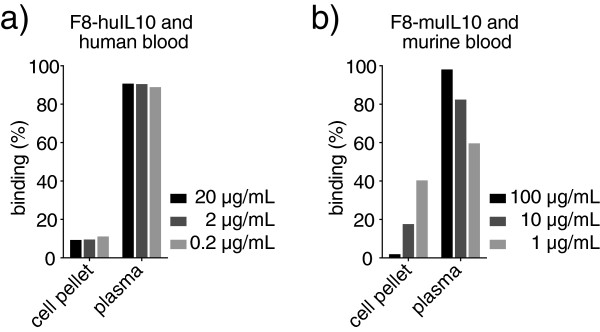
**Incubation experiment of radiolabeled immunocytokines with blood, analyzed by a centrifugation-based assay. (a)** Different concentrations of F8-huIL10 were incubated with fresh human blood. **(b)** Different concentrations of F8-muIL10 were incubated with fresh mouse blood. In both cases, inhibitors of coagulation were used. After a centrifugation step and separation of plasma from the cell pellet, radioactivity was counted and expressed as percent of binding.

Immunocytokines are expected to exert their therapeutic activity by extravasation from blood vessels at sites of disease, followed by antibody binding to its cognate antigen and by a prolonged interaction of the cytokine moiety with leukocytes. Since the binding of IL-10 to its receptor on blood cells could potentially inhibit extravasation and disease-targeting activity, we measured the ability of radioiodinated preparations of F8-huIL10 and F8-muIL10 to interact with blood cells by a centrifugation-based assay. The two fusion proteins were incubated with human and murine blood, respectively, for 10 minutes at various concentrations. A centrifugation step followed by radioactivity counting in plasma and in the cellular pellet allowed one to determine the fraction of unbound immunocytokine. Figure 8 shows that ~90% of F8-huIL10 is not bound to blood cells at concentrations as low as 0.2 μg/ml, while F8-muIL10 revealed ~40% binding to blood at a concentration of 1 μg/ml.

## Discussion

We have described and characterized a murine version of etanercept and a chimeric version of F8-IL10, which represent useful tools for the preclinical testing of these two therapeutic modalities, that are based on TNF inhibition and on the targeted delivery of an anti-inflammatory immunocytokine, respectively.

Murine versions of etanercept have previously been reported [13,19,20], but these articles did not provide full information about the cloning, purification and *in vitro* characterization of the fusion proteins. Barck and colleagues reported that their version of mTNFRII-Fc showed significant treatment effect in a murine model of collagen-induced arthritis at a concentration of 1 mg/kg (approximately 20 μg/mouse), upon analysis of computer-assisted tomography data [19]. In our study, we see *in vivo* activity in the 10 to 30 μg/mouse dose range.

In a first *in vivo* experiment, we used a full collagen induction protocol (see Methods). This led to a rapid onset of arthritis, with 30% of the mice developing explosive disease (that is, moved within 1 day from score 0 to score >3; excluded from therapy). The mice included in the therapy experiment showed strong progression in arthritic score, which was only inhibited by the combination therapy (F8-huIL10 plus muTNFR-Fc). In contrast to what has been previously reported by our group in a less aggressive model of arthritis [8], F8-huIL10 did not show a significant therapeutic effect, when used as a single agent. In a second *in vivo* experiment the booster injection was reduced to 80%, resulting in a milder development of the disease with only 10% of the mice excluded from therapy. In this case, TNF blockade (*P* = 0.0035), F8-huIL10 (*P* = 0.0561) and the combination treatment (*P* = 0.0023) inhibited arthritis progression upon day 8 when compared with saline (Figure 4f). We have previously demonstrated that the therapeutic activity of targeted IL-10 (for example, F8-huIL10 or L19-huIL10) is superior to the one of IL-10 fused to an antibody of irrelevant specificity in the mouse [7,8].

No correlation could be found between treatment groups and the generation of collagen-specific antibodies (Additional file 8). Furthermore, within the same mouse, healthy and inflamed structures could be observed in different paws, which prevented a homogeneous comparison of leukocyte infiltration in different treatment groups (Additional file 9).

Since one of the goals of our investigation was the assessment of immunogenic reactions using the fully human and the chimeric versions of F8-IL10, MAFAs were measured using BIAcore technology (Figure 5). The results show that chimerization did not reduce protein immunogenicity. Human and murine IL10 share 73% amino acid identity, whereas an alignment of corresponding germline V segments of antibody variable genes displays 40 to 80% amino acid identity between the two species [21-23]. The variability of CDR3 regions further contributes to sequence diversity between human and murine antibodies.

The issue as to whether immunocytokines can be trapped in blood by cellular components has received some attention in the past [15,24-26]. Preferably, the immunocytokine should not bind to leukocytes in blood, as this could prevent its ability to localize on its cognate marker of disease at extravascular sites. Figure 8 shows a surprising difference in cellular trapping between F8-huIL10 incubated with human blood and F8-muIL10 incubated with mouse blood. This discrepancy may reflect a different abundance of IL10 receptors between mouse and human leukocytes. Reassuringly, the clinical-stage product F8-huIL10 was not blocked by blood cells at concentrations as low as 0.2 μg/ml.

Dekavil (F8-huIL10) has shown selective targeting of arthritic lesions and inhibited progression of established disease in mice with collagen-induced arthritis [8] and is now being investigated in a phase 1b clinical trial in patients with arthritis in combination with methotrexate [11]. The results of this preclinical study provide a rationale for the combined use of Dekavil with TNF blockers, in line with previous reports about the combination of TNF blockade and IL-10 [4,27]. Previously, the combined use of TNF blockade with other anti-inflammatory biological agents (for example, combination of etanercept with anakinra) was found to be associated with increased safety risks in humans, including serious infections (0% for etanercept alone, 3.7 to 7.4% for etanercept plus anakinra), injection site reactions and neutropenia [28].

However, the excellent safety profile of recombinant human IL10 (Tenovil™) [5] and the fact that no signs of toxicity were observed in mice treated with muTNFR-Fc and F8-huIL10 suggest that a combination of etanercept with Dekavil may indeed be clinically feasible.

## Conclusions

The murine analogues of etanercept and of F8-huIL10 represent useful research tools for the study of TNF blockade and targeted cytokine delivery in mouse models of RA and other inflammatory conditions. The results presented in this study provide a rationale for the combination of Dekavil with etanercept in patients with RA, who do not respond sufficiently to either of the two monotherapies. Incubation studies performed with radiolabeled immunocytokines and blood indicate that F8-huIL10 is not blocked by leukocytes at concentrations as low as 0.2 μg/ml and that this product is thus free to extravasate from blood vessels and to reach its cognate antigen at sites of disease.

## Abbreviations

CHO: Chinese hamster ovary; EDA: Extra-domain A of fibronectin; ELISA: Enzyme-linked immunosorbent assay; i.v.: Intravenously; IL: Interleukin; MAFA: Mouse anti-fusion protein antibody; PBS: Phosphate-buffered saline; PCR: Polymerase chain reaction; RA: Rheumatoid arthritis; rhuIL10: Recombinant human IL10; rmuIL10: Recombinant murine IL10; s.c.: Subcutaneously; scFv: Single-chain variable fragment; TNF: Tumor necrosis factor; TNFR: Tumor necrosis factor receptor.

## Competing interests

DN is a cofounder and shareholder of Philogen SpA (Siena, Italy), the company that owns the F8 antibody and developed Dekavil (F8-huIL10). The experiments of this article have been co-financed by Philochem AG (Otelfingen, Switzerland), a fully-owned company of the Philogen group, in the frame of a collaborative Swiss Federal KTI MedTech Project with ETH (Kommission für Technologie und Innovation).

## Authors’ contributions

FD participated in designing the study, performed the cloning, production and characterization of the two fusion proteins, was responsible for the animal experiments and analysis of mouse plasma, and assisted in writing the manuscript. KS helped with designing the study and contributed to the animal experiments. TH helped with the animal experiments and performed the biodistribution experiment. DN designed the study, supervised the experiments and prepared the manuscript. All authors read and approved the final manuscript.

## Supplementary Material

Additional file 1 **Complete sequence of muTNFR-Fc.** The sequence for murine TNFR (amino acids 23 to 258) was directly fused to the murine Fc fragment (amino acids 98 to 324), containing the hinge, CH2 and CH3 regions. At the N-terminus a signal sequence (SS) was added. By *Hind*III and *Not*I double digest the insert was included into the mammalian cell-expression vector pcDNA3.1(+). Click here for file

Additional file 2 **Complete sequence of F8-muIL10.** The sequence for murine IL10 (amino acids 19 to 178) was appended at the C-terminus of the F8 antibody in diabody format, with a five-amino-acid linker between variable heavy chain (V_H_) and variable light chain (V_L_) and a 15-amino-acid linker ((SSSSG)_3_) between the antibody and IL-10. At the N-terminus a signal sequence (SS) was added. By *Nhe*I and *Not*I double digest the insert was included into the mammalian cell-expression vector pcDNA3.1(+). Click here for file

Additional file 3 **Replicate of analysis of cytokine levels in mice with full collagen-induced arthritis.** For each sample and cytokine, measurements were repeated on a different day in order to have an independent replicate of the assay. At the end of the therapy 13 different cytokine concentrations were measured in plasma using multiplex bead-based flow cytometry. Data points of cytokine concentrations above detection level are represented in a scatter plot with the mean ± standard error of the mean (*n* = 7). Standard curves defined with positive control samples (Additional file 6) were used to generate a level of quantification (LOQ; dotted red line). Click here for file

Additional file 4 **Replicate of analysis of cytokine levels in mice with reduced collagen-induced arthritis.** For each sample and cytokine, measurements were repeated on a different day in order to have an independent replicate of the assay. At the end of the therapy 13 different cytokine concentrations were measured in plasma using multiplex bead-based flow cytometry. Data points of cytokine concentrations above detection level are represented in a scatter plot with the mean ± standard error of the mean (n=10). Standard curves defined with positive control samples (Additional file 6) were used to generate a level of quantification (LOQ; dotted red line). Click here for file

Additional file 5 **Standard curves defined with positive control samples.** A mixture of standard cytokines was prepared according to the supplier’s protocol (eBioscience) and serially diluted. To determine cytokine concentration, a fluorescence-activated cell sorting analysis was performed on a BD FACS Canto and data evaluated with FlowCytomix Pro 3.0 software (eBioscience), generating the standard curves. Manually a level of quantification (LOQ) was superimposed on these standard curves (dotted green line). Click here for file

Additional file 6 **Standard curves defined with positive control samples for the repetition experiment.** A mixture of standard cytokines was prepared according to the supplier’s protocol (eBioscience) and serially diluted. To determine cytokine concentration, a fluorescence-activated cell sorting analysis was performed on a BD FACS Canto and data evaluated with FlowCytomix Pro 3.0 software (eBioscience), generating the standard curves. Manually a level of quantification (LOQ) was superimposed on these standard curves (dotted green line). Click here for file

Additional file 7 **Analysis of MAFA response.** (a) The flow rate over the sensor surface was 30 μl/minute for 3 minutes and the initial slope was calculated using the following equation: (response 60 seconds after the start of binding – response at baseline) / 60 seconds. (b) Initial slope of plasma samples and positive controls. Samples were passed over the two different flow cells coated with F8-huIL10 (black bars) or F8-muIL10 (grey bars) and the initial slope was calculated. Ab, antibody. Click here for file

Additional file 8 **Anti type-II collagen antibodies.** Titers of bovine type-II collagen-specific total IgG, IgG1 and IgG2a antibodies were determined using standard ELISA techniques. Briefly, 96-well plates were coated with 5 μg/ml bovine collagen II solution (Chondrex, Inc., Redmond, WA, USA) overnight at 4°C. Plasma samples diluted 1:800 were incubated for 1 hour and detected with horseradish peroxidase conjugated anti-mouse IgG, IgG1 and IgG2a antibodies (Santa Cruz, Heidelberg, Germany). Healthy*, mice were immunized but did not show any signs of inflammation. Click here for file

Additional file 9 **Immunohistochemical and immunofluorescence analysis of paw sections.** At the end of the therapy, paws from different therapy groups were frozen in cryoembedding medium (Neg50; Thermo Scientific, Wohlen, Switzerland) and stored at -80°C for analysis. (a) Hematoxylin and eosin staining of healthy and inflamed paw. Paw cryosections (10 μm) were fixed in ice-cold acetone and stained with hematoxylin solution Gill No. 2 (Sigma Aldrich) and alcoholic eosin Y solution (Sigma Aldrich). 10× magnification; scale bars = 50 μm. (b) Immunofluorescence analysis of infiltrating cells. Cryosections (10 μm) were fixed in ice-cold acetone and immunofluorescence staining was performed using primary antibodies against the following antigens: rat anti-mouse CD45 (leukocytes, 1:200; BD Bioscience), rabbit anti-asialo GM1 (NK cells, 1:4,000; Wako Pure Chemical Industries, Tokyo, Japan), rat anti-mouse CD4 (CD4^+^ cells, 1:50; BioXCell, West Lebanon, NH, USA) and rat anti-mouse CD8 (CD8^+^ cells, 1:50; BioXCell). Donkey anti-rat AlexaFluor488 (1:200; Invitrogen) and goat anti-rabbit AlexaFluor488 (1:200; Invitrogen) were used as secondary antibodies for detection. Sections were mounted with fluorescent mounting medium (Dako, Baar, Switzerland) and analyzed with an Axioskop2 mot plus microscope (Zeiss, Feldbach, Switzerland). The following scoring system was used for semiquantitative analysis of infiltrating cells: 0 = negative, 1 = single areas of positive cells with weak to moderate staining intensity, 2 = single areas of positive cells with strong staining intensity or disseminated positivity with weak to moderate staining intensity, 3 = large areas of positive cells within the whole tissue section with moderate to strong staining intensity. 10× magnification; scale bar = 50 μm (for all images). (c) Results of semiquantitative analysis for the different infiltrating cells (*n* = 2, F8-muIL10 *n* = 1). Healthy*, mice were immunized but did not show any signs of inflammation. Click here for file
