# Supplementary material for: Murine analogues of etanercept and of F8-IL10 inhibit the progression of collagen-induced arthritis in the mouse
Source: Arthritis Res Ther. 2013 Sep 27;15(5):R138. doi: 10.1186/ar4319 (PMC3978877; doi:10.1186/ar4319)
Supplement: Additional file 1 — Complete sequence of muTNFR-Fc. The sequence for murine TNFR (amino acids 23 to 258) was directly fused to the murine Fc fragment (amino acids 98 to 324), containing the hinge, CH2 and CH3 regions. At the N-terminus a signal sequence (SS) was added. By HindIII and NotI double digest the insert was included into the mammalian cell-expression vector pcDNA3.1(+). [file ar4319-S1.pdf]

HindIII - signal sequence (SS) - muTNFR - muFc (Hinge; CH2; CH3) - **Stop** - NotI

CCCAAGCTTGTCGACCATGGGCTGGAGCCTGATCCTCCTGTTCCTCGTCGCTGTGGCTACAGGTGTGCACTCGGTGCCCG
CCCAGGTTGTCTTGACACCCTACAAACCGGAACCTGGGTACGAGTGCCAGATCTCACAGGAATACTATGACAGGAAGGCT
CAGATGTGCTGTGCTAAGTGTCCTCCTGGCCAATATGTGAAACATTTCTGCAACAAGACCTCGGACACCGTGTGTGCGGA
CTGTGAGGCAAGCATGTATACCCAGGTCTGGAACCAGTTTCGTACATGTTTGAGCTGCAGTTCTTCCTGTACCACTGACC
AGGTGGAGATCCGCGCCTGCACTAAACAGCAGAACCGAGTGTGTGCTTGCGAAGCTGGCAGGTACTGCGCCTTGAAAACC
CATTCTGGCAGCTGTCGACAGTGCATGAGGCTGAGCAAGTGCGGCCCTGGCTTCGGAGTGGCCAGTTCAAGAGCCCCAAA
TGGAAATGTGCTATGCAAGGCCTGTGCCCCAGGGACGTTCTCTGACACCACATCATCCACTGATGTGTGCAGGCCCCACC
GCATCTGTAGCATCCTGGCTATTCCCGGAAATGCAAGCACAGATGCAGTCTGTGCGCCCGAGTCCCCAACTCTAAGTGCC
ATCCCAAGGACACTCTACGTATCTCAGCCAGAGCCCACAAGATCCCAACCCCTGGATCAAGAGCCAGGGCCCAGCCAAAC
TCCAAGCATCCTTACATCGTTGGGTTCAACCCCCATTATTGAACAAAGTACCAAGGGTGGCGTGCCCAGGGATTGTGGTT
GTAAGCCTTGCATATGTACAGTCCCAGAAGTATCATCTGTCTTCATCTTCCCCCCAAAGCCCAAGGATGTGCTCACCATT
ACTCTGACTCCTAAGGTCACGTGTGTTGTGGTAGACATCAGCAAGGATGATCCCGAGGTCCAGTTCAGCTGGTTTGTAGA
TGATGTGGAGGTGCACACAGCTCAGACAAAACCCCGGGAGGAGCAGTTCAACAGCACTTTCCGTTCAGTCAGTGAACTTC
CCATCATGCACCAGGACTGGCTCAATGGCAAGGAGTTCAAATGCAGGGTCAACAGTGCAGCTTTCCCTGCCCCCATCGAG
AAAACCATCTCCAAAACCAAAGGCAGACCGAAGGCTCCACAGGTGTACACCATTCCACCTCCCAAGGAGCAGATGGCCAA
GGATAAAGTCAGTCTGACCTGCATGATAACAGACTTCTTCCCTGAAGACATTACTGTGGAGTGGCAGTGGAATGGGCAGC
CAGCGGAGAACTACAAGAACACTCAGCCCATCATGGACACAGATGGCTCTTACTTCGTCTACAGCAAGCTCAATGTGCAG
AAGAGCAACTGGGAGGCAGGAAATACTTTCACCTGCTCTGTGTTACATGAGGGCCTGCACAACCACCATACTGAGAAGAG
CCTCTCCCACTCTCCTGGTAAA**TAG**CTTAATGAGCGGCCGCAAAAGGAAAA
