# Supplementary material for: Murine analogues of etanercept and of F8-IL10 inhibit the progression of collagen-induced arthritis in the mouse
Source: Arthritis Res Ther. 2013 Sep 27;15(5):R138. doi: 10.1186/ar4319 (PMC3978877; doi:10.1186/ar4319)
Supplement: Additional file 2 — Complete sequence of F8-muIL10. The sequence for murine IL10 (amino acids 19 to 178) was appended at the C-terminus of the F8 antibody in diabody format, with a five-amino-acid linker between variable heavy chain (VH) and variable light chain (VL) and a 15-amino-acid linker ((SSSSG)3) between the antibody and IL-10. At the N-terminus a signal sequence (SS) was added. By NheI and NotI double digest the insert was included into the mammalian cell-expression vector pcDNA3.1(+). [file ar4319-S2.pdf]

NheI - signal sequence (SS) - F8(diabody) - linker - muIL10 - **Stop** - NotI

CCCGCTAGCGTCGACCATGGCTGGAGCCTGATCCTCCTGTTCCTCGTCGCTGTGGCTACAGGTGTGCACTCGGAGGTGC
AGCTGTTGGAGTCTGGGGGAGGCTTGGTACAGCCTGGGGGGTCCCTGAGACTCTCCTGTGCAGCCTCTGGATTCACCTTT
AGCCTGTTTACGATGAGCTGGGTCCGCCAGGCTCCAGGGAAGGGGCTGGAGTGGGTCTCAGCTATTAGTGGTAGTGGTGG
TAGCACATACTACGCAGACTCCGTGAAGGGCCGGTTCACCATCTCCAGAGACAATTCCAAGAACACGCTGTATCTGCAAA
TGAACAGCCTGAGAGCCGAGGACACGGCCGTATATTACTGTGCGAAAAGTACTCATTTGTATCTTTTTGACTACTGGGGC
CAGGGAACCCTGGTCACCGTCTCGAGTGGCGGTAGCGGAGGGGAAATTGTGTTGACGCAGTCTCCAGGCACCCTGTCTTT
GTCTCCAGGGGAAAGAGCCACCCTCTCCTGCAGGGCCAGTCAGAGTGTTAGCATGCCGTTTTTAGCCTGGTACCAGCAGA
AACCTGGCCAGGCTCCCAGGCTCCTCATCTATGGTGCATCCAGCAGGGCCACTGGCATCCCAGACAGGTTCAGTGGCAGT
GGGTCTGGGACAGACTTCACTCTCACCATCAGCAGACTGGAGCCTGAAGATTTTGCAGTGTATTACTGTCAGCAGATGCG
TGGTCGGCCGCCGACGTTCGGCCAAGGGACCAAGGTGGAAATCAAATCTTCCTCATCGGGTAGTAGCTCTTCCGGCTCAT
CGTCCAGCGGCAGCAGGGGCCAGTACAGCCGGGAAGACAATAACTGCACCCACTTCCCAGTCGGCCAGAGCCACATGCTC
CTAGAGCTGCGGACTGCCTTCAGCCAGGTGAAGACTTTCTTTCAAACAAAGGACCAGCTGGACAACATACTGCTAACCGA
CTCCTTAATGCAGGACTTTAAGGGTTACTTGGGTTGCCAAGCCTTATCGGAAATGATCCAGTTTTACCTGGTAGAAGTGA
TGCCCCAGGCAGAGAAGCATGGCCCAGAAATCAAGGAGCATTTGAATTCCCTGGGTGAGAAGCTGAAGACCCTCAGGATG
CGGCTGAGGCGCTGTCATCGATTTCTCCCCTGTGAAAATAAGAGCAAGGCAGTGGAGCAGGTGAAGAGTGATTTTAATAA
GCTCCAAGACCAAGGTGTCTACAAGGCCATGAATGAATTTGACATCTTCATCAACTGCATAGAAGCATACATGATGATCA
AAATGAAAAGC**TAG**GCGGCCGCAAAAGGAAA
