# Supplementary figures and images for: Murine analogues of etanercept and of F8-IL10 inhibit the progression of collagen-induced arthritis in the mouse
Source: Arthritis Res Ther. 2013 Sep 27;15(5):R138. doi: 10.1186/ar4319 (PMC3978877; doi:10.1186/ar4319)

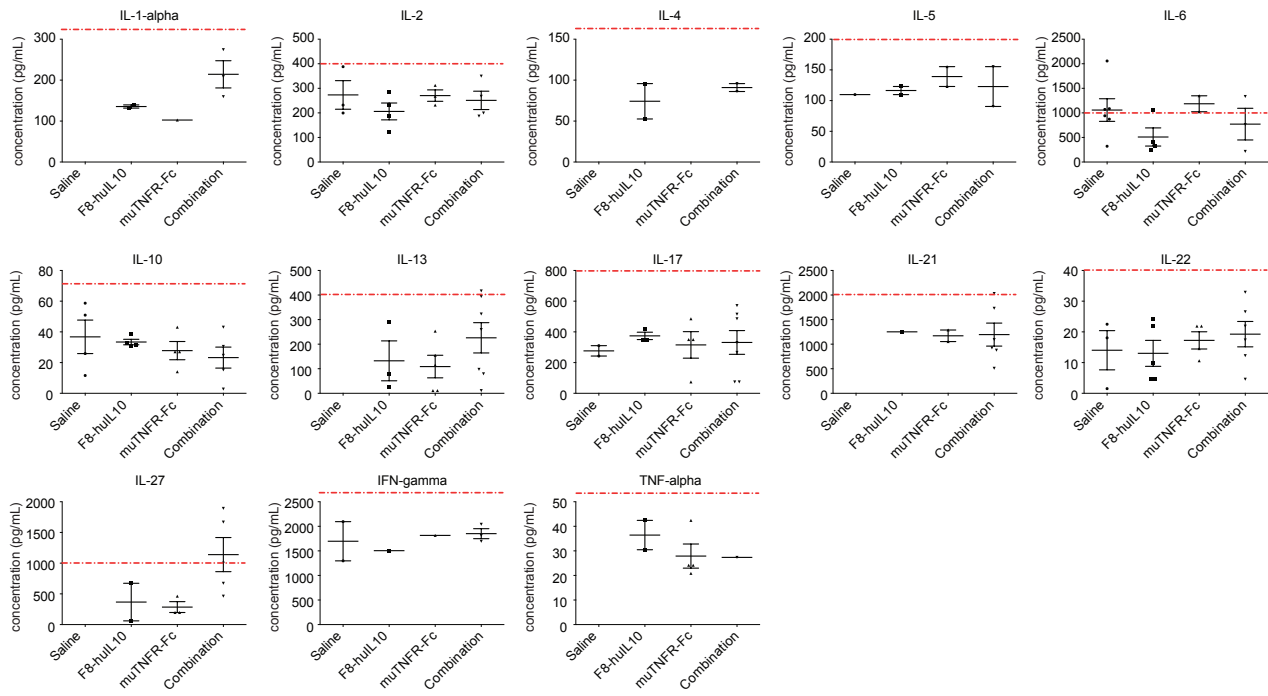

Supplement: Additional file 3 — Replicate of analysis of cytokine levels in mice with full collagen-induced arthritis. For each sample and cytokine, measurements were repeated on a different day in order to have an independent replicate of the assay. At the end of the therapy 13 different cytokine concentrations were measured in plasma using multiplex bead-based flow cytometry. Data points of cytokine concentrations above detection level are represented in a scatter plot with the mean ± standard error of the mean (n = 7). Standard curves defined with positive control samples (Additional file 6) were used to generate a level of quantification (LOQ; dotted red line). [file ar4319-S3.pdf]

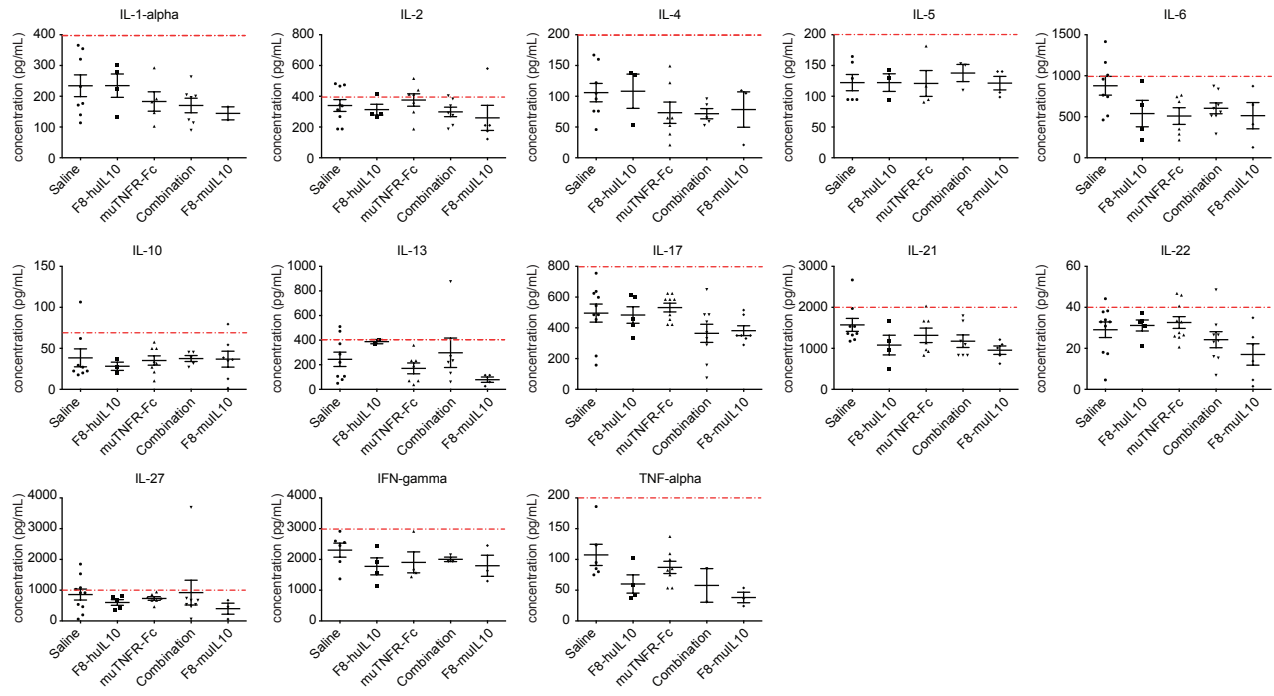

Supplement: Additional file 4 — Replicate of analysis of cytokine levels in mice with reduced collagen-induced arthritis. For each sample and cytokine, measurements were repeated on a different day in order to have an independent replicate of the assay. At the end of the therapy 13 different cytokine concentrations were measured in plasma using multiplex bead-based flow cytometry. Data points of cytokine concentrations above detection level are represented in a scatter plot with the mean ± standard error of the mean (n=10). Standard curves defined with positive control samples (Additional file 6) were used to generate a level of quantification (LOQ; dotted red line). [file ar4319-S4.pdf]

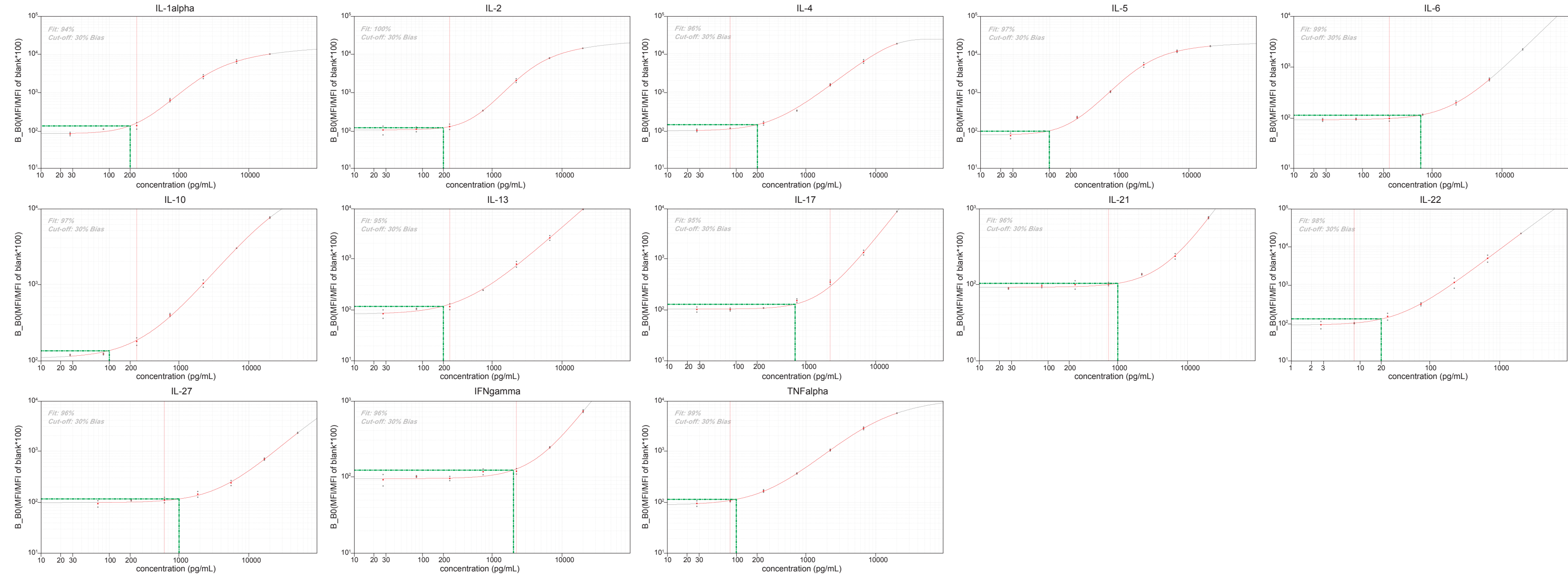

Supplement: Additional file 5 — Standard curves defined with positive control samples. A mixture of standard cytokines was prepared according to the supplier’s protocol (eBioscience) and serially diluted. To determine cytokine concentration, a fluorescence-activated cell sorting analysis was performed on a BD FACS Canto and data evaluated with FlowCytomix Pro 3.0 software (eBioscience), generating the standard curves. Manually a level of quantification (LOQ) was superimposed on these standard curves (dotted green line). [file ar4319-S5.pdf]

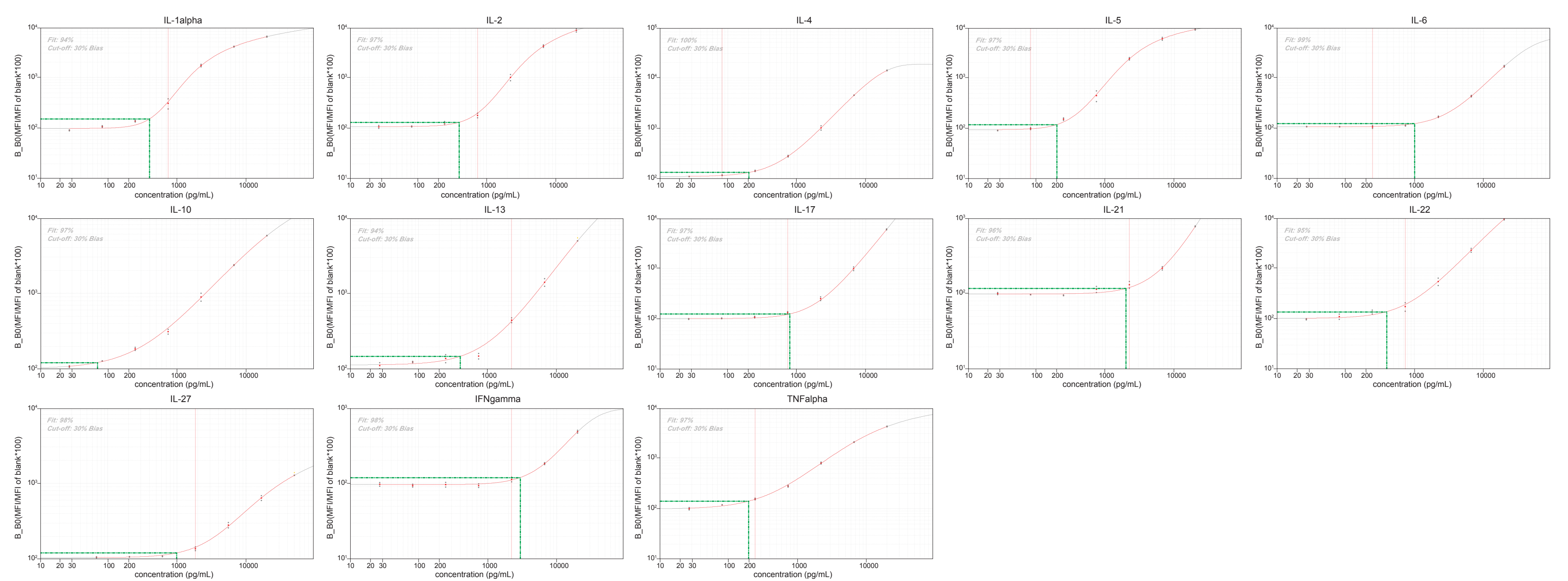

Supplement: Additional file 6 — Standard curves defined with positive control samples for the repetition experiment. A mixture of standard cytokines was prepared according to the supplier’s protocol (eBioscience) and serially diluted. To determine cytokine concentration, a fluorescence-activated cell sorting analysis was performed on a BD FACS Canto and data evaluated with FlowCytomix Pro 3.0 software (eBioscience), generating the standard curves. Manually a level of quantification (LOQ) was superimposed on these standard curves (dotted green line). [file ar4319-S6.pdf]

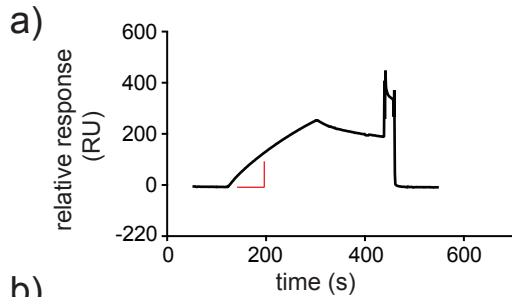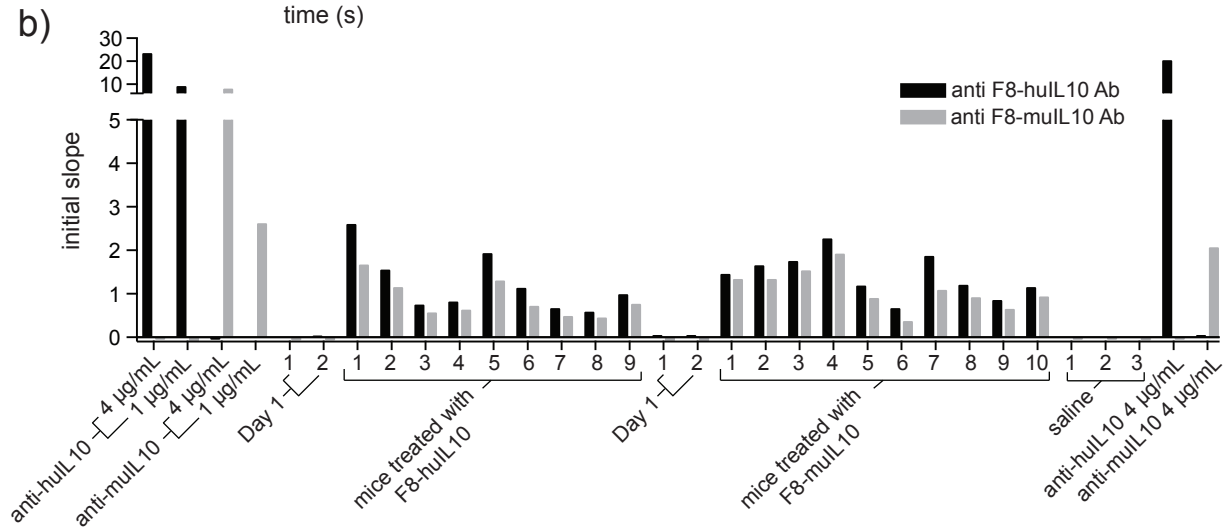

Supplement: Additional file 7 — Analysis of MAFA response. (a) The flow rate over the sensor surface was 30 μl/minute for 3 minutes and the initial slope was calculated using the following equation: (response 60 seconds after the start of binding – response at baseline) / 60 seconds. (b) Initial slope of plasma samples and positive controls. Samples were passed over the two different flow cells coated with F8-huIL10 (black bars) or F8-muIL10 (grey bars) and the initial slope was calculated. Ab, antibody. [file ar4319-S7.pdf]

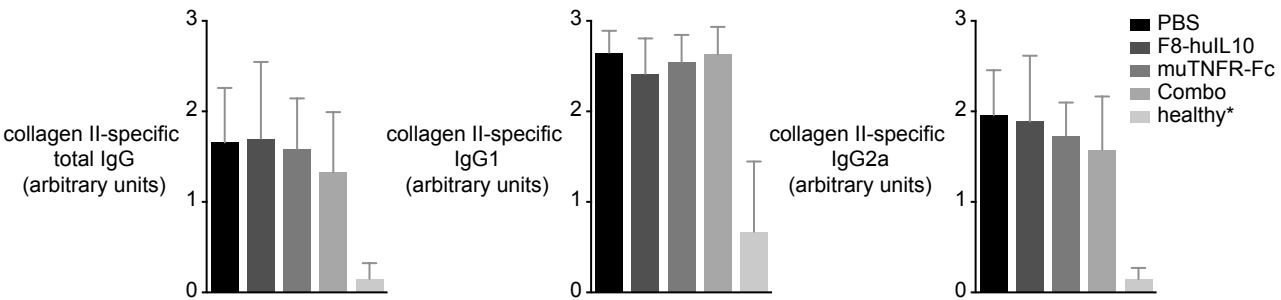

Supplement: Additional file 8 — Anti type-II collagen antibodies. Titers of bovine type-II collagen-specific total IgG, IgG1 and IgG2a antibodies were determined using standard ELISA techniques. Briefly, 96-well plates were coated with 5 μg/ml bovine collagen II solution (Chondrex, Inc., Redmond, WA, USA) overnight at 4°C. Plasma samples diluted 1:800 were incubated for 1 hour and detected with horseradish peroxidase conjugated anti-mouse IgG, IgG1 and IgG2a antibodies (Santa Cruz, Heidelberg, Germany). Healthy*, mice were immunized but did not show any signs of inflammation. [file ar4319-S8.pdf]

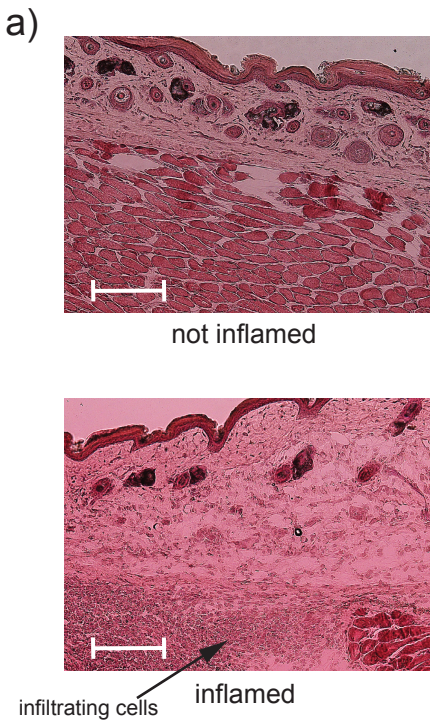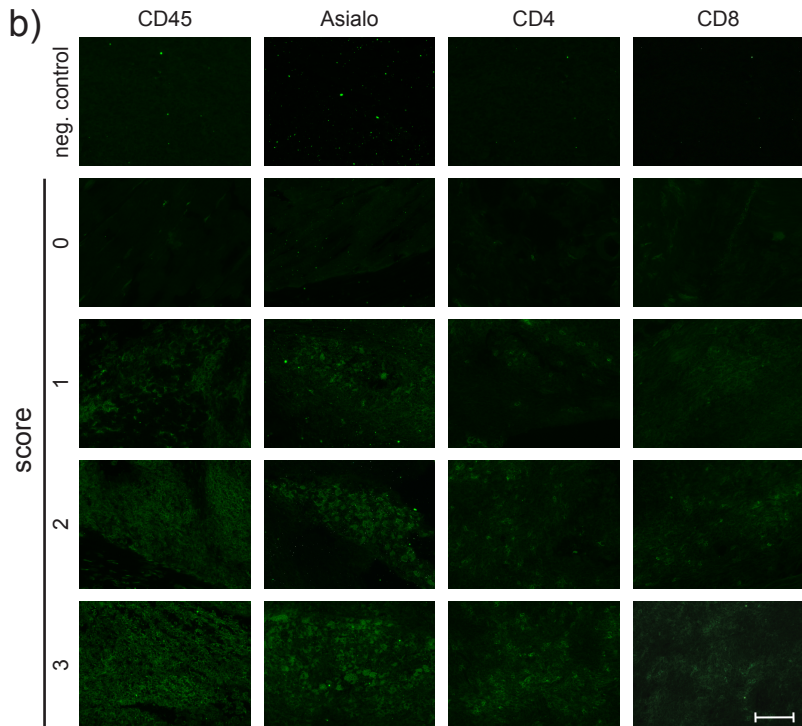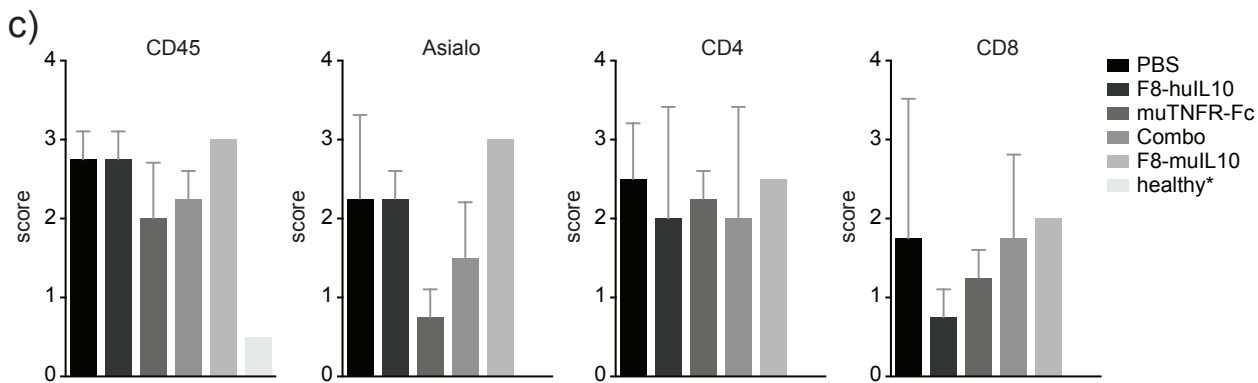

Supplement: Additional file 9 — Immunohistochemical and immunofluorescence analysis of paw sections. At the end of the therapy, paws from different therapy groups were frozen in cryoembedding medium (Neg50; Thermo Scientific, Wohlen, Switzerland) and stored at -80°C for analysis. (a) Hematoxylin and eosin staining of healthy and inflamed paw. Paw cryosections (10 μm) were fixed in ice-cold acetone and stained with hematoxylin solution Gill No. 2 (Sigma Aldrich) and alcoholic eosin Y solution (Sigma Aldrich). 10× magnification; scale bars = 50 μm. (b) Immunofluorescence analysis of infiltrating cells. Cryosections (10 μm) were fixed in ice-cold acetone and immunofluorescence staining was performed using primary antibodies against the following antigens: rat anti-mouse CD45 (leukocytes, 1:200; BD Bioscience), rabbit anti-asialo GM1 (NK cells, 1:4,000; Wako Pure Chemical Industries, Tokyo, Japan), rat anti-mouse CD4 (CD4+ cells, 1:50; BioXCell, West Lebanon, NH, USA) and rat anti-mouse CD8 (CD8+ cells, 1:50; BioXCell). Donkey anti-rat AlexaFluor488 (1:200; Invitrogen) and goat anti-rabbit AlexaFluor488 (1:200; Invitrogen) were used as secondary antibodies for detection. Sections were mounted with fluorescent mounting medium (Dako, Baar, Switzerland) and analyzed with an Axioskop2 mot plus microscope (Zeiss, Feldbach, Switzerland). The following scoring system was used for semiquantitative analysis of infiltrating cells: 0 = negative, 1 = single areas of positive cells with weak to moderate staining intensity, 2 = single areas of positive cells with strong staining intensity or disseminated positivity with weak to moderate staining intensity, 3 = large areas of positive cells within the whole tissue section with moderate to strong staining intensity. 10× magnification; scale bar = 50 μm (for all images). (c) Results of semiquantitative analysis for the different infiltrating cells (n = 2, F8-muIL10 n = 1). Healthy*, mice were immunized but did not show any signs of inflammation. [file ar4319-S9.pdf]
